# Genetic landscape and macro-evolution of co-circulating Coxsackieviruses A and Vaccine-derived Polioviruses in the Democratic Republic of Congo, 2008-2013

**DOI:** 10.1371/journal.pntd.0007335

**Published:** 2019-04-19

**Authors:** Serge Alain Sadeuh-Mba, Hugo Kavunga-Membo, Marie-Line Joffret, Riziki Yogolelo, Marie Claire Endegue-Zanga, Maël Bessaud, Richard Njouom, Jean-Jacques Muyembe-Tamfu, Francis Delpeyroux

**Affiliations:** 1 Virology Service, Centre Pasteur of Cameroon, Yaounde, Centre region, Cameroon; 2 Virology Department, Institut National de Recherche Biomédicale, Kinshasa, Democratic Republic of Congo; 3 Biology of Enteric Viruses Unit, Institut Pasteur, Paris, France; 4 INSERM U994 Unit, INSERM, Paris, France; Oxford University Clinical Research Unit, VIET NAM

## Abstract

Enteroviruses (EVs) are among the most common viruses infecting humans worldwide but only a few Non-Polio Enterovirus (NPEV) isolates have been characterized in the Democratic Republic of Congo (DR Congo). Moreover, circulating vaccine-derived polioviruses (PVs) [cVDPVs] isolated during multiple outbreaks in DR Congo from 2004 to 2018 have been characterized so far only by the sequences of their VP1 capsid coding gene. This study was carried to i) investigate the circulation and genetic diversity of NPEV and polio vaccine isolates recovered from healthy children and Acute Flaccid Paralysis (AFP) patients, ii) evaluate the occurrence of genetic recombination among EVs belonging to the *Enterovirus C* species (including PVs) and iii) identify the virological factors favoring multiple emergences of cVDPVs in DR Congo. The biological material considered in this study included i) a collection of 91 Sabin-like PVs, 54 cVDPVs and 150 NPEVs isolated from AFP patients between 2008 and 2012 in DR Congo and iii) a collection of 330 stool specimens collected from healthy children in 2013 in the Kasai Oriental and Maniema provinces of DR Congo. Studied virus isolates were sequenced in four distinct sub-genomic regions 5’-UTR, VP1, 2C^ATPase^ and 3D^pol^. Resulting sequences were compared through comparative phylogenetic analyses. Virus isolation showed that 19.1% (63/330) healthy children were infected by EVs including 17.9% (59/330) of NPEVs and 1.2% (4/330) of type 3 Sabin-like PVs. Only one EV-C type, EV-C99 was identified among the NPEV collection from AFP patients whereas 27.5% of the 69 NPEV isolates typed in healthy children belonged to the EV-C species: CV-A13 (13/69), A20 (5/69) and A17 (1/69). Interestingly, 50 of the 54 cVDPVs featured recombinant genomes containing exogenous sequences in at least one of the targeted non-structural regions of their genomes: 5’UTR, 2C^ATPase^ and 3D^pol^. Some of these non-vaccine sequences of the recombinant cVDPVs were strikingly related to homologous sequences from co-circulating CV-A17 and A20 in the 2C^ATPase^ region as well as to those from co-circulating CV-A13, A17 and A20 in the 3D^pol^ region. This study provided the first evidence uncovering CV-A20 strains as major recombination partners of PVs. High quality AFP surveillance, sensitive environmental surveillance and efficient vaccination activities remain essential to ensure timely detection and efficient response to recombinant cVDPVs outbreaks in DR Congo. Such needs are valid for any epidemiological setting where high frequency and genetic diversity of Coxsackieviruses A13, A17 and A20 provide a conducive viral ecosystem for the emergence of virulent recombinant cVDPVs.

## Author summary

The strategy of the Global Polio Eradication Initiative is based on the surveillance of patients suffering from Acute Flaccid Paralysis (AFP) and mass vaccination with live-attenuated vaccine strains of polioviruses (PVs) in endemic areas. However, vaccine strains of PVs can circulate and replicate for a long time when the vaccine coverage of the population is low. Such prolonged circulation and replication of vaccine strains of PVs can result to the emergence of circulating vaccine-derived polioviruses [cVDPVs] that are as virulent as wild PVs. In this study, we performed the molecular characterization of a large collection of 377 virus isolates recovered from paralyzed patients between 2008 and 2012 in DR Congo and healthy children in 2013 in the Kasai Oriental and Maniema provinces of DR Congo. We found that the genetic diversity of enteroviruses of the species *Enterovirus C* is more important than previously reported. Interestingly, 50 of the 54 cVDPVs featured recombinant genomes containing exogenous sequences of the 2C ATPase and/or 3D polymerase coding genes acquired from co-circulating Coxsackieviruses A13, A17 and A20. Coxsackieviruses A20 strains were identified for the first time as major partners of genetic recombination with co-circulating live-attenuated polio vaccine strains.

Our findings highlight the need to reinforce and maintain high quality surveillance of PVs and efficient immunization activities in order to ensure early detection and control of emerging cVDPVs in all settings where high frequency and diversity of Coxsackieviruses A13, A17 and A20 have been documented.

## Introduction

Acute flaccid paralysis (AFP) is a clinical syndrome induced in humans by infectious agents (bacterial or viral), non-infectious causes (trauma, metal toxicity and metabolic disorders) or post-infectious autoimmune conditions (eg: Guillian Barre syndrome) [1, 2]. Viral etiologic agents frequently involved in AFP include enteroviruses (EVs), flaviviruses, herpesviruses, rabies virus and tick borne encephalitis virus [3, 4].

Enteroviruses (EVs) are members of the genus *Enterovirus*, family *Picornaviridae*. Based on their phylogenetic relationships, the (sero)types of EVs infecting humans are classified into 7 species, *Enterovirus A* to *D* (EV-A to -D) and *Rhinovirus A* to *C* (RV-A to -C). Further 7 species infect pigs and sheeps (EV-G), cows (EV-E and -F), nonhuman primates (EV-H, EV-J and EV-L) and rodents (EV-K) [5].

The most recognized severe EV-induced disease is paralytic poliomyelitis, specifically caused by polioviruses (PVs). The Global Polio Eradication Initiative, based AFP surveillance and mass vaccination with live-attenuated PVs, has led to the reduction of the incidence of wild PV associated poliomyelitis from an estimated 350,000 cases in 1988 to only 22 cases in 2017: 14 in Afghanistan and 8 in Pakistan [6, 7]. In particular, wild PV2 and wild PV3 have disappeared worldwide [6, 8, 9]. However, this outstanding progress towards eradication is threatened by the emergence of circulating vaccine-derived PVs (cVDPVs) [6].

Since 2000, many poliomyelitis outbreaks associated to cVDPVs have been reported mainly in developing countries including those in Madagascar in 2001–2002 and 2005 [10, 11], Nigeria (2005–2015) [12] and Democratic Republic of Congo (DR Congo) [13].

After the original interruption of wild PV transmission in DR Congo from 2001 to 2005, outbreaks of wild PV1 and wild PV3 were reported in 10 of the 11 provinces the DR Congo from 2006 to 2011 as result of importations from neighboring Angola [14, 15]. Moreover, DR Congo has been stricken by the second largest cVDPV induced poliomyelitis outbreak in Africa; behind the top ranking Nigeria [7, 13, 16]. Indeed, type 2 cVDPV were repeatedly isolated from 2004 to 2012 [13, 17]. Epidemic investigation and appropriate immunization responses stopped wild PV associated outbreaks, with the most recent confirmed wild PV case reported in Maniema province in 2011 [13, 15]. Nonetheless, five years apart from the large 2008–2012 outbreak, DR Congo has recently been affected by cVDPV2 associated poliomyelitis outbreaks in 2017 and 2018 [18, 19]. This suggests that the viral ecosystem and epidemiological conditions of DR Congo may be favorable to multiple cVDPV emergence.

Most cVDPVs isolated during poliomyelitis outbreaks that have been fully characterized were shown to be recombinants between Oral Polio vaccine (OPV) strains and non-polio EV-C [20–23]. In particular, non-polio EV-C sequences of the genome of type 2 and type 3 cVDPVs were shown to derive from co-circulating Coxsackieviruses A13 (CV-A13) and CV-A17 and possibly CV-A11 ancestors [21–23]. Using genetic engineering techniques, non-polio EV-C derived exogenous sequences of the recombinant cVDPVs were shown to contribute to their phenotypic characteristics including pathogenicity [24–26]. From pioneer studies in Madagascar, it has been suggested that an enteroviral ecosystem marked by a high rate and diversity of some CV-A of the EV-C species provide a conducive virological environment to cVDPV emergence and circulation in a population under low OPV coverage.

In addition to Madagascar, recent studies have uncovered extensive circulation and diversity of CV-A of the EV-C species in other sub-Saharan countries including Cameroon, Central African Republic and Chad [27–29]. However, frequent cVDPVs emergence and circulation have not been documented in these countries likely because of the relatively higher rates of OPV coverage [30]. Thus, it would be of great interest to investigate the circulation and genetic landscape of CV-A13, CV-A17 and other CV-A variants in countries known to have been affected by, and are thus at risk of, large cVDPV outbreaks. Such countries include Nigeria and DR Congo [7, 12, 13]. In the other hand, only very few NPEV isolates originating from DR Congo have been analyzed by molecular characterization [31–35] despite their common occurrence observed during routine poliomyelitis surveillance [15, 36].

This study was designed to i) investigate the genetic drift of OPV strains isolated from healthy children and AFP patients, ii) search for potential silent circulation of cVDPVs and/or wild PVs and iii) evaluate the diversity and occurrence of genetic recombination among co-circulating EV-C, including cVDPVs and non-polio EV-C in DR Congo where multiple independent cVDPV emergences have been documented.

## Results

### Virus isolation and differentiation of polioviruses from healthy children

Virus isolation from the collection of stool specimens from healthy children showed an overall isolation rate at 19.1% (63/330) on the RD, L20B and HEp-2C cell lines with heterogeneous isolation profiles (Table 1). Among the 330 children enrolled, 17.9% (59/330) were positive for NPEVs while 1.2% (4/330) was positive for PVs (Table 1). All PV isolates identified from four children were found to be Sabin-like type 3 by intratypic differentiation (ITD) test.

**Table 1:** Virus isolation from the stool specimens from healthy children.

Isolates^a^		Stool specimens (N = 330)	
Virus	Types of infected cell lines	Number of CPE-positive samples (n)^b^	Isolation rate (%)
PVs	RD and L20B	4	1.2
NPEVs	RD	14	4.2
NPEVs	HEp-2c	26	7.9
NPEVs	RD and HEp-2c	19	5.8
**Total NPEVs**		59	17.9
**Total EVs**		63	19.1

^a^ PVs, polioviruses; NPEVs, Non-polio enteroviruses; EVs, enteroviruses

^b^ CPE, cytopathic effects; Given the fact that 19 specimens produced isolates on both cell lines, a total of 78 NPEV isolates were obtained.

Stool specimens from nineteen out the 59 NPEV-infected healthy children showed a CPE in both RD and HEp-2c cell cultures. Thus, overall 78 isolates derived from healthy children were considered as NPEVs (Table 1) and subjected to molecular typing.

### Genetic drift of OPV strains and variability of cVDPV isolates

**Fig 1:**
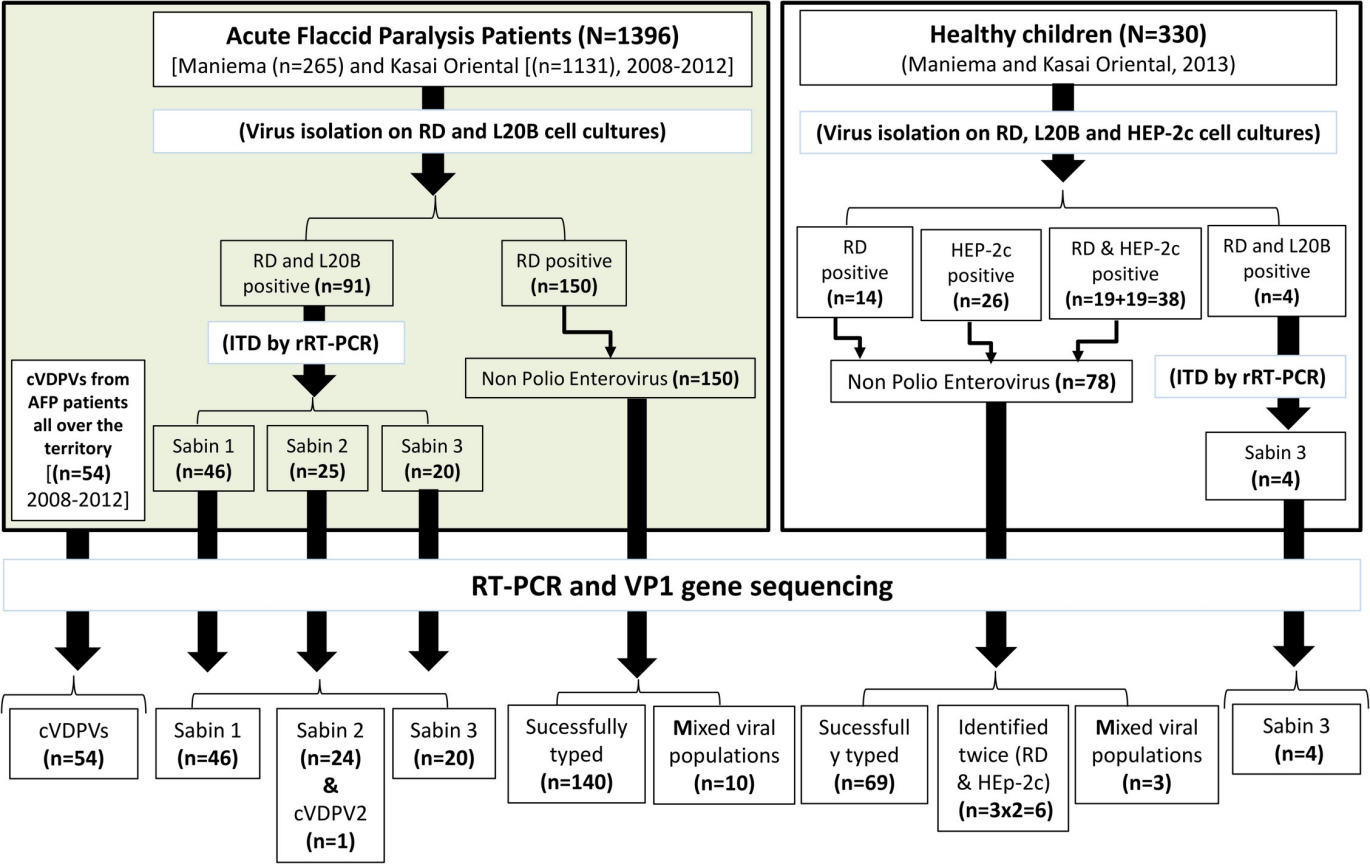
Summary of the studied collections of stools, virus isolates and overall results of cell cultures and molecular typing. Circulating vaccine-derived polioviruses (cVDPVs) were obtained from Acute Flaccid Paralysis (AFP) cases originating all over the territory. They were isolated and typed using the same techniques as for Sabin-like polioviruses. ITD, intratypic differentiation; rRT-PCR, real-time RT-PCR.

To search for potential genetic drift among the 95 studied Sabin-like isolates, including 91 from AFP patients and 4 from healthy children (Fig 1), we compared the full-length VP1 sequence of each isolate with homologous sequence of the corresponding Sabin strain.

We found that all 46 Sabin type 1 and 24 Sabin type 3 isolates from AFP cases and healthy children were OPV-like with ≤ 2 nt substitutions compared to original vaccine Sabin types 1 and 3, respectively (Fig 1). Concerning the 25 isolates from AFP patients that were identified as Sabin type 2 by real time RT-PCR based Intratypic Differentiation (ITD), 24 were found to have ≤ 2nt substitutions compared to Sabin type 2. The remaining one was actually a type 2 cVDPV genetically linked to the 2010 outbreak in the Maniema province. Though the failure of the ITD assay in routine setting was exceptional, it is of great interest to carry out the sequencing of the VP1 gene of all field Sabin 2 isolates identified by ITD in the current context where type 2 vaccine have been withdrawn from the polio immunization strategy worldwide.

Interestingly, there was no evidence of silent circulation of wild PV and cVDPV among healthy children. Sequence analyses also showed that OPV isolates from healthy children had accumulated ≤ 3 mutations in their full-length VP1 capsid coding gene. The limited divergence of these Sabin-like isolates is consistent with the fact that they originated from children who had received OPV immunization less than 2 weeks before stool sampling.

### Characterization of the polymerase coding gene of OPV strains

In order to search for OPV/non-polio EV-C recombination in the absence of VP1 sequence divergence, Restriction Fragment Length Polymorphism (RFLP) assay using restriction enzymes HinfI, DpnII, DdeI, and RsaI was carried out on the 929-bp generated from the 3D-3’UTR region of all Sabin-like isolates.

All field Sabin-like PVs showed RFLP profiles similar to those of homotypic or heterotypic reference Sabin strains. These data indicate that all OPV strains from the field were either non-recombinant OPV strains or intertypic recombinants between OPV strains as it has been extensively documented.

### Molecular typing of NPEV isolates

In this study, NPEVs were typed using the sequences of the 5’ half of the VP1 gene. Amplicons covering partial or complete sequence VP1 gene of all 228 NPEVs analyzed, including 78 isolates from 59 healthy children and 150 isolates from AFP patients were successfully amplified and sequenced. The VP1 sequence of 209 isolates could be determined while the remaining 13 isolates (03 from healthy children and 10 from AFP patients) could not be typed due to unexploitable sequencing output due to electropherograms with superimposed peaks (Table 2). Such sequencing results were interpreted as the effect of co/super-infection by at least two different strains and corresponding isolates were not further investigated. Concerning healthy children in particular, co/super-infection with at least two EV types were documented in 16 of the 59 (27.1%) EV-infected healthy children. Thirteen of such co/super-infection could be successfully typed since their HEp-2c and RD derived isolates contained different virus types.

**Table 2:** Distribution of non-polio EV types and species among healthy children and acute flaccid paralysis patients in the Kasai Oriental and Maniema provinces of the Democratic Republic of Congo.

	Virus isolates					
	Healthy children (N = 78 isolates)		AFP patients (N = 150 isolates)		Total (N = 228 isolates)	
types/species	n*	%	n	%	n	%
**HEV-A**						
EV-A76			1	0.7	1	0.5
***All EV-A isolates***				1	0.7	1	0.5
**EV-B**						
CV-B1			2	1.4		2	1.0
CV-B2	1	1.4	3	2.1	4	1.9
CV-B3*	16	23.2	3	2.1	19	9.1
CV-B4*	12	17.4	3	2.1	15	7.2
CV-B5	1	1.4	6	4.3	7	3.3
CV-B6	2	2.9	4	2.9	6	2.9
E-1	2	2.9	6	4.3	8	3.8
E-2			2	1.4	2	1.0
E-3	3	4.3	7	5.0	10	4.8
E-4			1	0.7	1	0.5
E-6	1	1.4	15	10.7	16	7.7
E-7			6	4.3	6	2.9
E-11*	2	2.9	24	17.1	26	12.4
E-12			9	6.4	9	4.3
E-13	1	1.4	6	4.3	7	3.3
E-14			7	5.0	7	3.3
E-19			1	0.7	1	0.5
E-20			4	2.9	4	1.9
E-21			1	0.7	1	0.5
E-24	4	5.8	5	3.6	9	4.3
E-25	2	2.9			2	1.0
E-29			4	2.9	4	1.9
E-31			2	1.4	2	1.0
E-33			4	2.9	4	1.9
EV-B69	2	2.9	1	0.7	3	1.4
EV-B74			1	0.7	1	0.5
EV-B75	1	1.4	7	5.0	8	3.8
EV-B93			2	1.4	2	1.0
EV-B97			1	0.7	1	0.5
***All EV-B isolates***	**50**	72.5	**137**	97.9	187	89.5
**EV-C**						
CV-A13	13	18.8			13	6.2
CV-A17	1	1.4			1	0.5
CV-A20	5	7.2			5	2.4
EV-C99			1	0.7	1	0.5
***All EV-C isolates***	**19**	27.5	**1**	0.7	20	9.6
**EV-D**						
EV-D111			1	0.7	1	0.5
***All EV-D isolates***	** **	0.0	**1**	0.7	1	0.5
**All isolates typed**	**69**	**100.0**	**140**	**100.0**	**209**	**100.0**
Mixing strains	3	3.8	10	6.7	13	5.7
Same isolate on RD and HEP-2c	6	7.7	na	na	na	na
**All isolates**	**78**	**100.0**	**150**	**100.0**	**228**	**100.0**

N, total number of isolates; n, number of isolates in each type; %, percentage among all typed isolates

*, Among the 19 healthy children from whom 19 RD and 19 HEp-2c derived isolates were obtained, thirteen showed heterotypic isolates while the remaining six contained the same virus type from RD and HEp-2c cell lines. The number of EV types specified by a star includes only one of the homotypic isolates recovered from the same child using RD and HEp-2c cell lines, respectively.

na, not applicable

Other 3 co/super-infections remained uncharacterized because both HEp-2c and RD isolates showed unexploitable electropherograms with superimposed peaks (Table 3). Most characterized co/super-infections in healthy children was found to be mixtures of heterotypic EV-B isolates (Table 3).

**Table 3:** Summary of mono and co(super)-infection among non-polio enterovirus-infected healthy children.

	Non-polio enterovirus positive healthy children	
Infections	N	%
**Mono-infections**		
**Total mono-infection**	**43**	**72.9**
**Co-infections**		
Characterized co-infections*		
CV-B3 and CV-B4	3	
CV-B3 and E-24	1	
CV-B4 and untyped strain mixture	1	
CV-B4 and CV-A13	1	
CV-B3 and E-1	1	
E-3 and E-13	1	
CV-B6 and EV-B69	1	
CV-B5 and E-24	1	
CV-B3 and E-6	1	
CV-B3 and CV-B6	1	
E-1 and EV-B69	1	
**Total characterized co-infections**	**13**	
**Total uncharacterized co-infections (strain mixture)**	**3**	
**Total co-infections**	**16**	**27.1**
**Total infection**	**59**	**100**

*CV-B, Coxackievirus B; E-1, Echovirus 1; EV-B, Enterovirus B; n, number of children; %, percentage among all isolates obtained.

### Diversity of non-polio enterovirus types from healthy children and paralyzed patients

Among the NPEV isolates obtained from healthy children 17 different EV types, including 14 EV-B and 3 EV-C types, were identified. The summary of strains distribution into EV types and species is provided in Table 2. The rate of EV-B types was as high as 72.5% (50/69). Accordingly, two EV-B types were found in all but one co/super-infections that could be characterized (Table 3). Interestingly, a relatively high rate of EV-C at 27.5% (19/69) was found among all typed isolates and CV-A13 was the most frequent type, accounting for more than half (13/19) of all typed isolates from healthy children (Table 2). Among the 19 studied EV-C isolates identified, 11 CV-A13 were from the Maniema province while 2 CV-A13, 1 CV-A17 and all 5 CV-A20 originated from the Kasai Oriental province. No isolate belonging to EV-A and -D was found among healthy children.

Concerning isolates obtained from AFP patients, almost all (137 of the 140 NPEVs that could be typed) belonged to the EV-B species while species EV-A, -C and -D were represented by one EV-A76, EV-C99 and EV-D111 isolates respectively (Table 2). As many as 28 different EV-B types were found and they comprised all EV-B types derived from healthy children, with the exception of Echovirus 25 (E-25) (Table 2). In contrast to healthy children, isolation of EV-C was as rare as the isolation of EV-A and -D. This could be associated to the fact that HEp-2c cells were not used during the routine surveillance of PVs.

### Phylogenetic relationships of the studied EV-A and -D isolates

**Fig 2:**
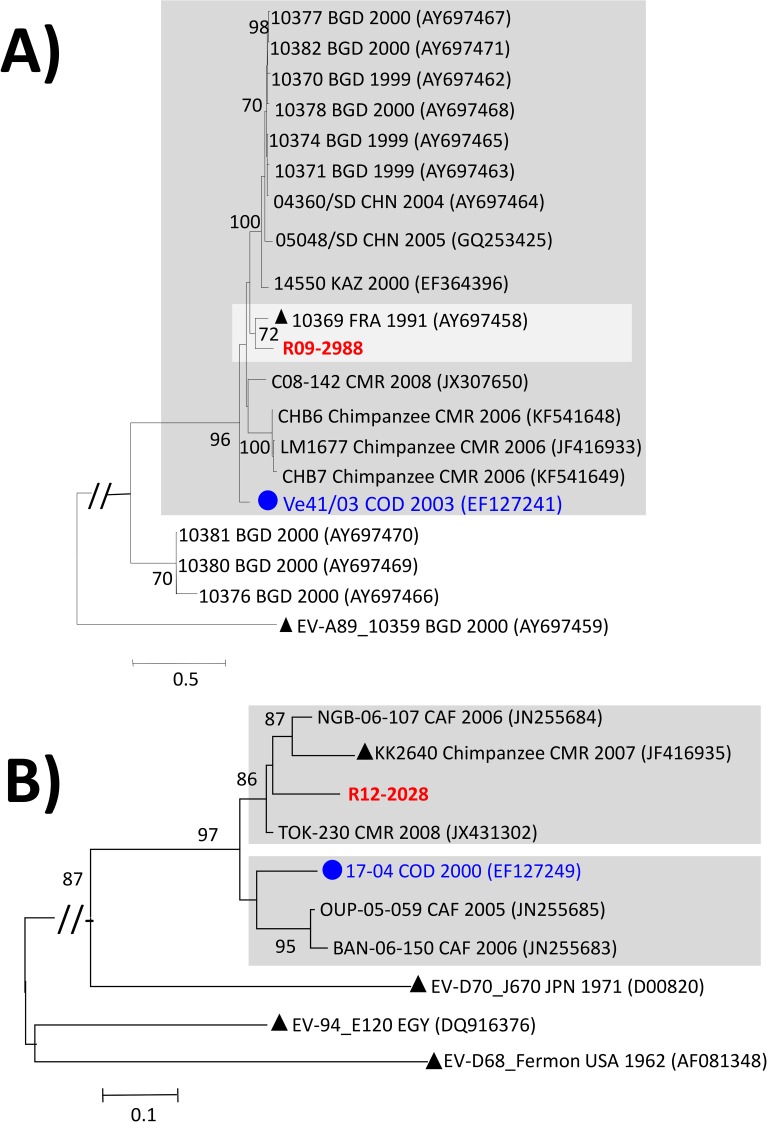
Phylogenetic relationships of the full-length VP1 sequences of the studied EV-A and -D. Maximum likelihood trees were inferred from the alignments of full-length VP1 sequences of EV-A76 using the GTR+G+I model of nucleotide substitution (A) and partial VP1 sequences of EV-D111 strains (nt 1–471 according to the VP1 sequence of the EV-D111 prototype strain KK2640) using the T92+G model of nucleotide substitution (B). Studied isolates are specified in bold red while isolates previously reported in DR Congo are highlighted by circles in bold blue. The year and country of isolation of each reference isolate are indicated, when known (BGD: Bangladesh; CAF: Central African Republic; CMR: Cameroon; CHN: China; COD: Democratic Republic of the Congo; EGY: Egypt; FRA: France; JPN, Japan; KAZ: Kazakhstan; USA: United-States of America). Prototype strains are highlighted by black triangles. For clarity, bootstrap values less than 70% have been omitted and the scale bars indicate nucleotide distance as substitutions per site. Isolates belonging to specific lineages commented in the main text are gathered in grey-shaded boxes.

The unique EV-A identified in this study, the EV-A76 R09-2988 isolated from an AFP patient in 2009, clustered with the EV-A76 prototype strain 10369 isolated in France in 1991. In contrast, it clustered distinctly from other EV-A76 isolates from Sub-Saharan Africa including the isolate 41–03 isolated in 2003 in DR Congo and more recent isolates originating from humans and chimpanzees in Cameroon [28, 37, 38] (Fig 2A).

As observed for EV-A76, the unique EV-D111 strain R12-2028 clustered, with strong bootstrap support, with human and chimpanzee derived EV-D111 originating from Cameroon and Central African Republic [28, 29, 37, 38]. However, it clustered distinctly from the lineage defined by the oldest EV-D111 strain 17–04 isolated from an AFP case in the DR Congo in 2000 [31] (Fig 2B).

### Genetic diversity of the studied EV-B isolates

This study revealed a tremendous genetic diversity of EV-B isolates in the DR Congo. As much as 29 of the 63 currently known EV-B types were found among all isolates identified. The most frequent virus type were E-11, followed by Coxsackievirus B3 (CV-B3), E-6 and CV-B4 represented by 26, 19, 16 and 15 isolates respectively (Table 2). However, CV-B3 and CV-B4 were strikingly more prevalent among healthy children whereas E-11 and E-6 were seemingly more frequent among AFP patients (Table 2). Several EV types recently discovered in Bangladesh and the United States [39, 40]; including EV-B74, EV-B75, EV-B93, and EV-B97; were also found.

### Genetic diversity of the studied EV-C isolates

**Fig 3:**
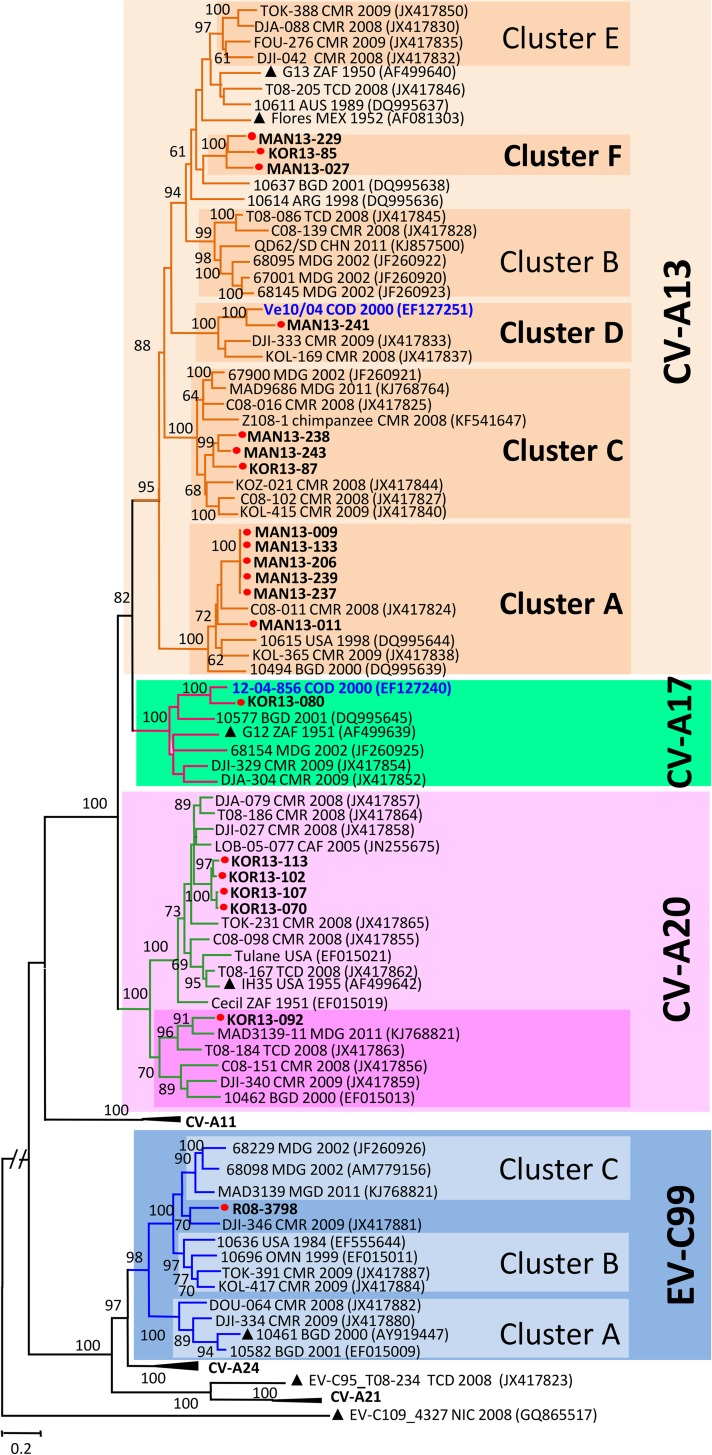
Phylogenetic relationships of the newly sequenced EV-C isolates from DR Congo. The Maximum likelihood phylogram was inferred from the alignment of full-length VP1 sequences of EV-C strains using the most complex GTR+G+I model of nucleotide substitution. Previously described isolates originating from DR Congo are indicated in bold blue while isolates from this study are highlighted by red circles. Isolates from healthy children are named with a 3 letter code indicating the province of origin (MAN: Maniema and KOR: Kasai Oriental) whereas the unique EV-C99 recovered from a paralyzed child is R08-3798. The year and country of isolation of each reference isolate are indicated, when known (ARG: Argentina; AUS: Australia; BGD: Bangladesh; CAF: Central African Republic; CMR: Cameroon; CHN: China; COD: Democratic Republic of the Congo; NIC: Nicaragua; MEX: Mexico; MDG: Madagascar; OMN: Oman; TCD: Chad; USA: United-States of America; ZAF: South Africa). In addition to the sequences of prototype strains, sequences displaying highest similarities with the studied isolates were retrieved from databases using NCBI BLAST search and included as references in the analyzed dataset. Prototype strains are highlighted by triangles. For clarity, most bootstrap values less than 60 have been omitted. The scale is shown at the bottom, as substitutions per site. Isolates belonging to the virus types and lineages commented in the main text are gathered in color-shaded boxes.

Full-length VP1 sequences of all studied non-polio EV-C isolates were unambiguously identified based on the recommended type assignment thresholds [41]. As expected, individual nt VP1 sequences of the study isolates segregated into type-specific clusters featuring strong bootstrap supports (Fig 3). Within the CV-A13, and A20 type groups, individual isolates segregated into several strongly supported clusters defining new or previously unknown genetic lineages.

As expected from our previous reports, the highest intra-typic genetic variability was featured by the most prevalent EV-C type CV-A13 (Fig 3). Studied CV-A13, including 11 from the Maniema province and 2 from the Kasai Oriental province (RDC13-085 and RDC13-087), segregated into three previously known clusters A, C and D [28, 29, 42] along with a candidate new lineage tentatively assigned as “cluster F” (Fig 3). This cluster F included three healthy children derived CV-A13 isolates: RDC13-027 and RDC13-229 originating respectively from the districts of Alunguli and Kindu in the Maniema province and isolate RDC13-085 from the district of Citenge in the Kasai Oriental province. The fact that these CV-A13 isolates were isolated from two distinct provinces from March to October 2013 indicates that the lineage defining the CV-A13 Cluster F circulates in the DR Congo.

No CV-A13 isolate from DR Congo fell in the cluster B which included CV-A13 strains originating from Cameroon, Central African Republic and Madagascar as well as in the cluster E defined exclusively by isolates originating from Cameroon (Fig 3). Within the Central African specific Cluster D, the studied CV-A13 RDC13-241 clustered with the previously characterized CV-A13 isolate Ve10/04 originating from an AFP case from DR Congo in 2000 [31]. Overall, our findings provide support to the fact that CV-A13 circulating in Sub-Saharan Africa is much more diversified than previously documented.

Within the CV-A20 defined group, all five studied CV-A20 isolated in Kasai Oriental province segregated into two lineages with strong bootstrap supports. Four isolates clustered together within the lineage defined by the CV-A20 prototype strain IH-35 while the remaining isolate fell in a separate cluster along with CV-A20 isolates from Sub-Saharan Africa and Bangladesh (Fig 3).

CV-A17 and EV-C99 types were represented by unique isolates recovered respectively from an AFP case in 2008 and a healthy child in 2013. The unique CV-A17 isolate clustered with the isolate 12-04-856 originating from an AFP case from DR Congo in 2000. The studied EV-C99 isolate RDC08-3790 from the district of Omendjadi in The Kasai Oriental province, clustered with EV-C99 isolate DJI-346 originating from Cameroon in 2009. Both isolates defined a potentially new lineage of EV-C99 (Fig 3).

### Genetic variability of cVDPV isolates

**Fig 4:**
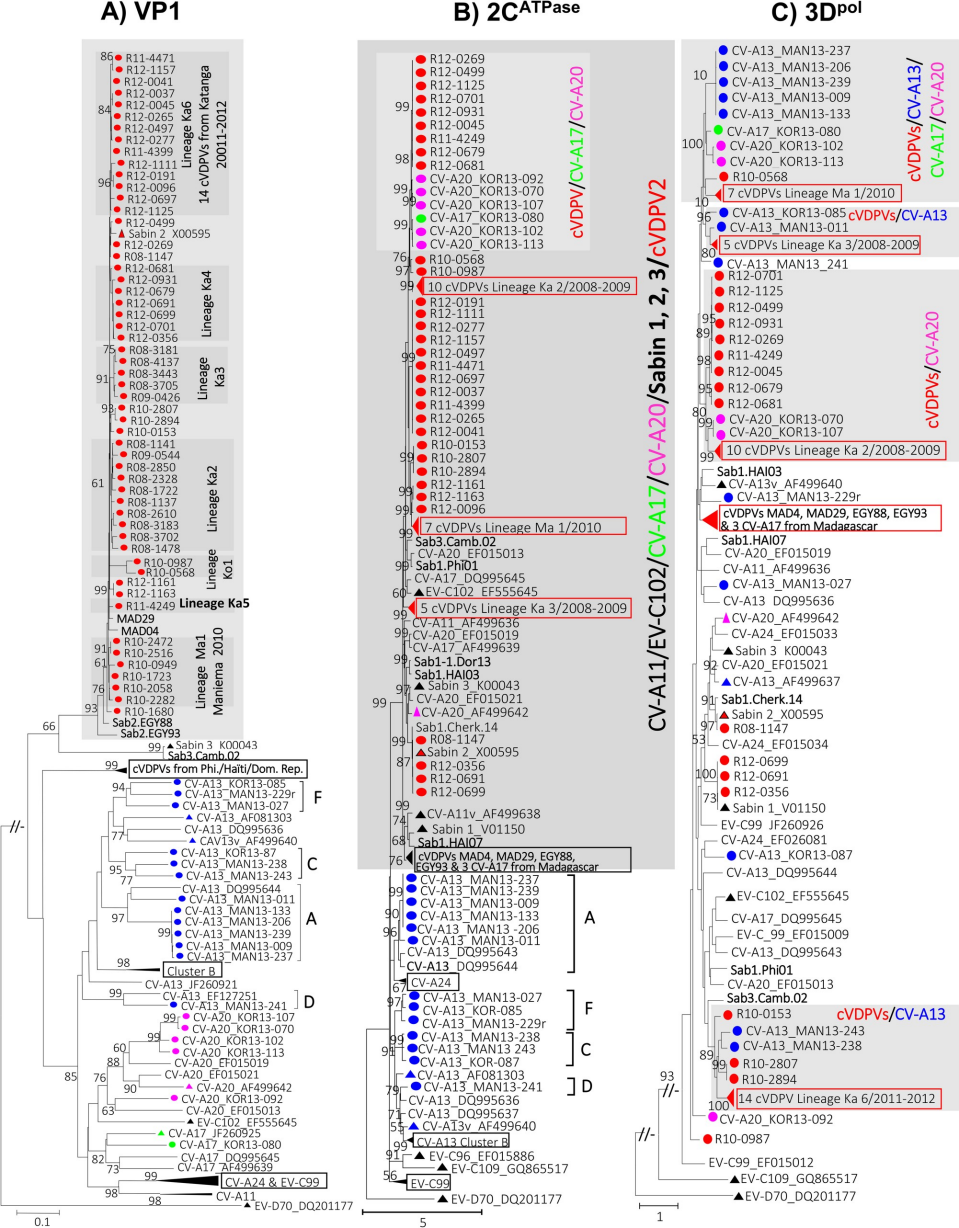
Phylogenetic trees depicting the genetic relationships between the nucleotide sequences derived from sub-genomic regions of species C enteroviruses. Maximum likelihood trees were inferred from specific alignments of partial nucleotide sequences of the VP1, 2C^ATPase^ and 3D^pol^ coding genes [nucleotide positions 2482 to 2953 for VP1 (A); 4123 to 4922 for 2C^ATPase^ (B); 6166 to 6837 for 3D^pol^ (C) according to the complete genome sequence of Sabin 2]. Branch lengths were calculated using the best-fit model of nucleotide substitution estimated with Smart Model Selection based on the Bayesian Information Criterion: GTR+G for the VP1 (A), and 3D^pol^ (C) regions and GTR+G+I for the 2C^ATPase^ (B) region. Phylogenetic lineages of CV-A13 are indicated on the VP1 (A) and 2C^ATPase^ (B) derived trees using letters A to F. The genetic distance is indicated with a scale bar at the bottom. Numbers at nodes correspond to the percentage of 1,000 bootstrap replicates supporting the distal cluster. Since databases available sequences of wild polioviruses as well as most non-polio enteroviruses species C originating from other epidemiologic context featured no peculiar relationships with the studied isolates, most of them were omitted from the final datasets. However, selected poliovirus strains isolated during type 1, 2 and 3 cVDPV-associated outbreaks in Egypt (Sab2.Egy88, Sab2.Egy93), Madagascar (MAD04 and MAD29), Haiti (Sab1.HAI03, Sab1.HAI07), Dominican Republic (Sab1.Dor13), Philippines (Sab1.Phi01) and Cambodia in 2002 (Sab3.Cam02) were included. Trees were oriented using the nucleotide sequences of Enterovirus D70 (EV-D70) as outgroup. cVDPV isolates obtained from paralyzed patients from 2008 to 2012 and Coxsackievirus A viruses isolated from healthy children in 2013 in the Maniema (MAN) and Kasai Oriental (KOR) provinces of DR Congo are highlighted with circles color-coded according virus types: red for cVDPV, blue for CV-A13, green for the unique CV-A17 and purple for CV-A20 isolates. cVDPV identifiers include: “R” standing for DR Congo; “two digit code” indicating the year of isolation (08, 2008; 09, 2009; 10, 2010; 11, 2011 and 12, 2012) and “four digit code” representing the serial number. For clarity, some clusters defined by homotypic virus isolates were collapsed: Phi., Philippines; Dom. Rep., Domican Republic; Eq. and ka., Equateur and Katanga provinces of DR Congo. EV-C types and/or cVDPV sequences featuring peculiar relationships discussed in the text are gathered in grey-shaded boxes.

A previous report had provided details about the phylogenetic relationships of the studied cVDPVs isolates, except those from the Katanga province in 2012 [13]. Two independent cVDPV emergence events were detected in Maniema and Kasai Orientale in 2008 while two concomitant cVDPV2 outbreaks (lineages Ka2 and Ka3) were documented in 2008 in Katanga where both outbreaks extended in 2009 (Fig 4A).

Several other cVDPV lineages associated to independent emergence events were documented in 2009–2010 in Kasai Occidental (lineage Ko1), Equateur, Maniema (including lineage Ma1), Kasai Oriental, and Katanga Provinces. Further cVDPV emergence events occurred in late 2011 and continued into 2012 with several cVDPVs detected in Katanga. cVDPVs isolated during the 2011–2012 outbreaks defined lineages Ka4 and Ka5 as previously reported; as well as lineage Ka6 identified in this study. Overall, the 42 studied cVDPVs from the Katanga province segregated into at least 5 distinct phylogenetic lineages (Fig 4A).

### Recombination between co-circulating cVDPVs and non-polio EV-C strains

In order to evaluate potential recombination between co-circulating EV-C isolates, including cVDPVs and non-polio EV-C, the genomes of 54 type 2 cVDPVs isolated from AFP patients between 2008 and 2010 in DR Congo and those of the 19 EV-C from healthy children were further characterized by the investigation of phylogenetic discrepancies between the 5’UTR, 2C^ATPase^ and 3D^pol^ regions and the reference VP1 region.

Whereas VP1 sequences segregated according to specific virus types and intratypic clusters or lineages, 2C^ATPase^ and 3D^pol^ derived sequences showed striking phylogenetic variations compared to VP1 based clustering thus suggesting extensive recombination (Fig 4).

**Fig 5:**
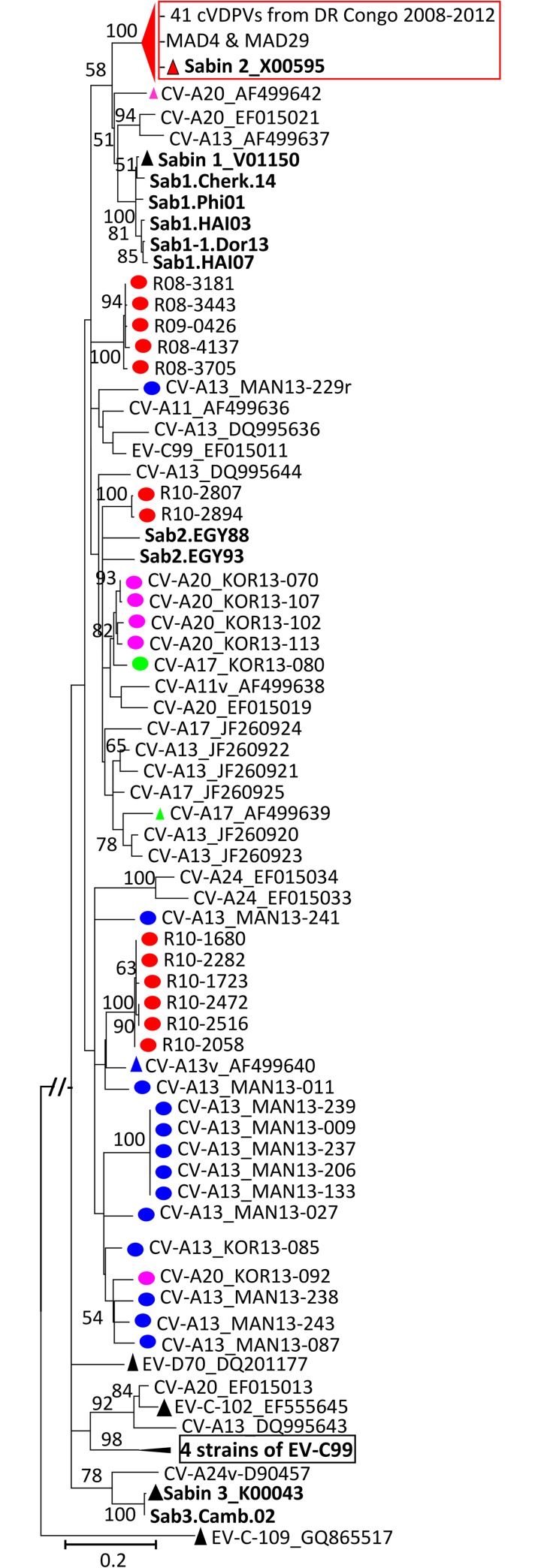
Phylogenetic trees depicting genetic relationships between nucleotide sequences derived from the 5’utr genomic regions of species C enteroviruses. The maximum likelihood tree was based on the alignment of partial nucleotide sequence of the 5’UTR region of the genome (nt 183 to 573 according the complete genome of the Sabin 2). Branch lengths were calculated using the best-fit model of nucleotide substitution GTR+G+I estimated with Smart Model Selection based on the Bayesian Information Criterion. Numbers at nodes correspond to the percentage of 1,000 bootstrap replicates supporting the distal cluster. For comparison, lineages depicted on the VP1-based reference phylogeny (Fig 4A), were highlighted here despite the fact that they were not supported by well-defined and bootstrap supported groups (Fig 4A). For comparison, the cluster defined by the 41 circulating vaccine-derived polioviruses featuring Sabin 2 related sequences were collapsed. Details about isolates’ labeling and the dataset considered are the same as specified in the legend of Fig 4.

For visibility, lineages depicted on the VP1-based reference phylogeny (Fig 4A) were associated to corresponding isolates on the 5’UTR region (Fig 5). From the 5’UTR sequences based tree (Fig 5), 41 of the 54 cVDPV2 showed Sabin 2 vaccine sequences as displayed by other cVDPV2 MAD4 and MAD29 from Madagascar. In contrast, 13 cVDPV isolates were recombinants, showing non-vaccine sequences in their 5’UTR region. However, none of the recombinants displayed close genetic relationships with non-polio EV-C in the 5’UTR region using the sequence dataset considered including EV-C isolates from DR Congo. These data confirm that PV/non-polio EV-C recombination involved the 5’UTR region of cVDPVs to some extent (Fig 4).

In contrast to the 5’UTR based phylogeny that revealed a limited number of PV/non-polio EV-C recombinants, as high as 50 of the 54 studied cVDPVs appeared to be recombinants having non-vaccine sequences in both 2C^ATPase^ and 3D^pol^ regions. All four remaining cVDPVs displayed Sabin 2 related 2C^ATPase^ sequences while they displayed Sabin 1 or Sabin 2 sequences in the 3D^pol^ region (Fig 4).

Interestingly, the phylogenetic pattern displayed by the studied strains in the 2C^ATPase^ region was more similar to that of the VP1 region with an outstanding exception. Indeed, a strongly supported clade clustered sequences derived from OPV, CV-A11, EV-C102, CV-A17, CV-A20 and cVDPVs strains. These PV and non-polio EV-C types fell in a particular clade within which intertypic recombination events are virtually common (Fig 4B). In particular, non-vaccine sequences of the genome of some recombinant cVDPVs from the Katanga province in 2012 were strikingly related to the CV-A17 and A20 derived from healthy children in the Kasai Oriental province in 2013 (Fig 4B).

It is noteworthy that, although CV-A13 was the most prevalent non-polio EV-C type, all 13 isolates were strikingly separate from the studied CV-A17, A20 and cVDPVs. Interestingly, even a certain level of intratypic segregation was displayed among CV-A13 lineages that fell outside the [PVs/CV-A11/CV-A17/CV-A20/EV-C102] clade (Fig 4B). These findings indicate that the probability and frequency of recombination between OPV strains and some non-polio EV-C type may not be exclusively governed by their relative frequency of co-infection in a given population. Intertypic genetic recombination affecting the 2C^ATPase^ region of EV-C strains may be under functional and/or mechanistic constraints that select recombination partners.

The 3D^pol^ region based phylogram also revealed close genetic relationships between cVDPVs, CV-A17 and A20 from distinct time and geographic ranges in DR Congo (Fig 4C). However, in contrast to 2C^ATPase^, 3D^pol^ region also showed consistent relatedness between the studied cVDPVs and CV-A13. This suggests that the overall sequence space provided by the tremendous diversity of CV-A13 and other CV-A in sub-Saharan Africa create a conducive viral environment favoring the macro-evolution of EV-C, including PVs. Differential constraints between the sub-genomic regions of EV-C types do not only select intertypic recombination partners. They also select genomic regions that can be exchanged through intertypic recombination. By so doing, such constraints shape the genetic and phenotypic properties of the EV-C recombinants that can emerge in their ecological niche.

## Discussion

EVs are among the most common viruses infecting humans worldwide [43], but data about the molecular epidemiology of EVs in Sub-Saharan Africa are still sparse. This study presents data on the circulation, genetic diversity and evolution of PVs and NPEVs among healthy children and AFP patients originating in DR Congo from 2008 to 2013.

During the study period, trivalent OPV (types 1, 2 and 3) were used for routine vaccination while bivalent and monovalent formulations were specifically used in response to prevalent strains of circulating PVs [44]. We found a low rate of PV strains among healthy children in the Kasai Oriental and Maniema provinces of DR Congo in 2013. Only 1.2% (4/330) of the stool specimens were positive for PV whilst a rate of at least 10% was expected based on previous studies in healthy children in some developing countries where OPV were also routinely used [10, 45, 46]. This low rate of silent PV circulation can be explained by the high polio vaccine coverage whose national average estimates varied from 64% in 2008 to 77% since 2009 [30] following immunization responses to cVDPV and wild PV associated outbreaks [15]. Accordingly, all PV isolates obtained in 2013 were identified as type 3 Sabin-like strains having ≤ 2 nt substitutions in their full-length VP1 sequences.

In contrast to PVs, we found high rate of NPEV infection among healthy children with an overall isolation rate at 17.9%. NPEV infection rate among healthy children was high compared to the annualized averages repeatedly reported from 2008 to 2013 among AFP patients. Indeed, annualized average rates of NPEV infection in the Kasai Oriental and Maniema provinces were consistently under the WHO-specified minimum at ≥ 10% [36]. The high rate of NPEV infection among healthy children compared to AFP patients could be primarily explained by the fact that HEp-2c cell culture was systematically used in the prospective virus isolation from healthy children. Accordingly, 26 of the 59 EV-infected children harbored NPEV that could be propagated solely in HEp-2c and not in RD cell cultures. Some studies have reported NPEV isolation rates above 30% in Cameroon [28] and 20% in Madagascar [23] using both RD and HEp-2c cell lines. Failure to isolate a significant proportion of circulating EV strains that are refractory to propagation on RD cell cultures is one of the possible explanation of the consistent failure to meet the WHO-specified indicator requiring that ≥ 10% of stool specimens submitted to the polio reference laboratory should have a NPEV isolated [47, 48]. Such observation has been documented in DR Congo [36].

### Inconsistency between the rates of EV-C among healthy children and Acute Flaccid Paralysis children

As expected from our previous studies, indicating that EV-C strains are more efficiently grown on HEp-2c than RD cell lines [28, 29, 49], isolation of EV-C from AFP patients was as rare as the isolation of EV-A and EV-D (Table 2). The absence of HEp-2c cells in the routine isolation algorithm of EVs from AFP cases has likely led to underestimation of the proportion of EV-C species in AFP cases. All EV-C types identified among healthy children in this study were recovered from HEp-2c cell cultures. In particular, CV-B4 and CV-A13 isolated from the child co/super-infected by EV-B and EV-C were respectively harvested on RD and HEp-2c cell cultures (Table 3). This suggests that the apparent difference between the isolation rate of EV-C in AFP cases and healthy children may be associated to the use of HEp-2c cells only for healthy children.

### Genetic diversity and phylogenetic relationships among EVs

As expected from our previous studies in sub-Saharan Africa based on RD and/or HEp-2c derived NPEV isolates [28, 29, 31, 49], isolation of virus types belonging the species EV-A and -D was very exceptional. Only an EV-A76 and an EV-D111 were identified from AFP patients. Despite the fact that EV-A76 and EV-D111 were discovered recently [37, 50], their respective diversification into at least two phylogenetic lineages indicates a certain level of circulation among human population in Central Africa and South Eastern Asia [28, 31, 50–52] (Fig 2). The segregation of EV-A76 isolates from this and a previous study in DR Congo into two distinct phylogenetic lineages could be explained either by the diversification or multiple introduction of that EV type in that country [28, 29, 31, 37].

Among EV-C isolates, the highest genetic diversity was displayed by the most frequent EV type CV-A13. We identified CV-A13 isolates belonging respectively to clusters A and C that have been previously reported in Madagascar and Central Africa [28, 29, 38, 42] and cluster D reported so far exclusively in Central Africa including RD Congo [28, 31]. Besides these previously known lineages, three studied isolates defined a new sub-Saharan CV-A13 cluster tentatively assigned as “cluster F”. As cluster E is made up exclusively by CV-A13 isolates from Cameroon [28], the newly identified cluster F was defined exclusively by isolates from RD Congo (Fig 4). Interestingly, the unique EV-C isolate from an AFP patient clustered with the Cameroonian strain DJI-346 that was previously suggested as potential new lineage among EV-C99 strains [28] (Fig 4). Altogether, this and our previous studies indicate that EV-C strains are highly prevalent and diversified in the sub-Saharan Africa.

### Recombination partners of OPV strains

We could not carry our field investigation (collection of stools from healthy children) in the Katanga province from where most studied cVDPVs originated because of acute security concerns. However, this study uncovered that cVDPVs and non-polio EV-C isolates from distinct time and geographical ranges were closely related in their 2C^ATPase^ and 3D^pol^ derived sequences sharing recent common ancestors (Fig 4). Reports from Madagascar and Cambodia had previously uncovered CV-A13 and -17, and probably CV-A11, as putative recombinant partners that provide exogenous sequences of the non-structural region of the genome of recombinant cVDPVs [22, 53]. Interestingly, the present study provides additional findings revealing CV-A20 as one of the major recombinant partners of OPV strains in DR Congo.

As previously observed in the studies in Madagascar [21, 22, 53], non-vaccine sequences of the genome of a number of recombinant cVDPVs could not be reliably assigned to particular EV-C types. Given that all studies conducted so far were based on the collection of isolates recovered from cell cultures, it cannot be excluded that the selection bias associated to virus isolation may have led to the missing of some EV-C types that are also recombination partners of OPV strains. Accordingly, some EV-C types, including CV-A1, CV-A19 and CV-A22, have been shown to be unable to grow in cell cultures [41]. Methodological approaches using random characterization directly from clinical and/or environmental samples could provide substantial data in an attempt to address the apparent gap in the EV-C diversity landscape. It is also noteworthy that cVDPVs characterized in this study have also co-circulated with wild PVs in DR Congo from 2008 to 2011. This implies that exhaustive analyses including wild PVs, non-polio EV-C and cVDPVs could have provided more insight into the macro-evolution of co-circulating EV-C types.

The methodology used for the study of recombination among EV-C types, based on the investigation of phylogenetic incongruences between different sub-genomic sequences, have been widely applied [23, 54–57]. In this study, the 2C^ATPase^ sequences featured clustering pattern nearly concordant with that of the VP1 region except within the divergent clade of [PV/CV-A11/CV-A13/CV-A17/CV-A20/EV-C102] (Fig 4B). These observations from field studies corroborate recent experimental studies indicating that genetic interaction between 2C^ATPase^ and viral capsid proteins is crucial for the morphogenesis of PVs [58, 59]. This could explain the fact that this and previous field studies have never identified EV-C/OPV recombinant having capsid genes derived from non-polio EV-C and at least one non-structural region derived from OPV strains. Such EV-C/OPV recombinants were successfully engineered but could not be propagated experimentally in cell cultures [60]. Along with recent studies, this study showed that at least some non-structural regions of the PV genome do not evolve as independently from the capsid region as originally thought. Apparently, functional and/or mechanistic constraints select the recombinant partners among co-circulating EV-C types, the symmetry of recombination between each other and genomic regions involved. This results to the selection of the fittest recombinants that emerge in natural settings.

### Virological factors of the emergence of circulating recombinant VDPVs

Extensive recombination events have been shown to shape the genetic and phenotypic properties of EVs [24, 25, 55, 56] and most cVDPVs have been shown to be recombinants emerging through recombination between PVs and other non-polio EV-C. These recombinant cVDPVs included 93.6% (50/54) of the studied cVDPVs originating from diverse time and geographic ranges in DR Congo (Figs 4 and 5). As discussed above, there was a relatively high rate and genetic diversity of EV-C strains circulating among healthy children in the Kasai Oriental and Maniema provinces of DR Congo in 2013. Thus, the diversity landscape of EVs in DR Congo provides an ideal setting for co-infection and subsequent recombination between co-circulating PVs and non-polio EV-C. Data from the study of cVDPVs from Cambodia and Madagascar had already demonstrated that CV-A13, CV-A17 and possibly CV-A11 are efficient recombination partners of PVs [10, 20, 22, 23]. This study provides substantial evidence indicating that CV-A20 is another privileged recombination partner of PVs. CV-A13 have been shown to be the closest non-polio EV-C related to PVs in the capsid coding region, followed by CV-A20, CV-A17 and CV-A11, respectively [60]. Seemingly, the relative frequency of recombination between PVs and individual CV-A of the EV-C species is inversely proportional to their P1 nt sequence divergence [60]. Together with previous reports, this study suggests that specific viral ecosystem, marked by high rate and high variability of CV-A13, CV-A17 as well as CV-A20, offers ideal virological conditions for the emergence of neurovirulent recombinant cVDPVs. That conducive virological factor combines with low polio vaccine coverage of the population to favor the emergence and circulation of virulent recombinant cVDPVs.

### Conclusion

DR Congo have been repeatedly stricken by cVDPV associated poliomyelitis outbreaks and the largest one occurred between 2008 and 2012. This study showed no evidence of silent circulation of cVDPV among healthy children in 2013 in the previously affected Kasai Oriental and Maniema provinces of DR Congo. In contrast, it revealed a relatively high rate and diversity of EV-C types in the healthy children enrolled. Findings from the analysis of recombination between PV and CV-A strains uncovered that: apart from CV-A13, CV-A17 and possibly CV-A11, CV-A20 is a privileged partner of recombination with type 2 OPV strains. This study provides further support to the hypothesis that the presence of a specific enteroviral ecosystem characterized by a high rate and diversity of at least some CV-A of the species EV-C, including CV-A11, A13, A17 and A20, is a conducive factor for the emergence and circulation of recombinant cVDPVs. Despite the fact that improved surveillance and efficient vaccination responses succeeded in stopping recombinant cVDPV associated outbreaks in 2012, DR Congo has been recently affected by cVDPV outbreaks in 2017 and 2018. This highlights the need to maintain high quality AFP and environmental surveillance systems to ensure early detection and response to cVDPV emergence in DR Congo and wherever high frequency and diversity of CV-A of the species EV-C have been documented.

## Methods

### Ethical considerations

This study involved a prospective enrollment of healthy children and a retrospective inclusion of cell culture isolates recovered from the stools of AFP patients analyzed at the WHO-accredited national reference laboratory for poliomyelitis at the Institut National de Recherche Biomédicale (INRB) within the framework of poliomyelitis surveillance in DR Congo. The study protocol was reviewed and approved by the Research Ethics Review Committee of the School of Public Health at the University of Kinshasa (approbation number ESP/CE/042/2012). All participants were ≤ 15 years and were enrolled in this study after the informed consent of their parents or legal guardians. Stool specimens and associated data were anonymously and confidentially handled.

### Field investigations

A total of 330 stool specimens from apparently healthy children ≤ 10 years originating from two provinces of DR of Congo were enrolled in this study. They comprised 168 specimens from the districts of Dibindi, Lukelenge, Tshishimbi, Lubilanji, Muya, Kansele, Bonzola, Diulu, Bipemba, Mpokolo, Citenge, Nzaba in the Kasaï Oriental province and 162 specimens from the districts of Alunguli, Kindu, Rva, Omata and Luama in the Maniema province. Stool specimens were collected from each participant along with demographic and epidemiological data including age, sex, site of enrollment and OPV immunization history (based both on vaccination card and/or parents’ declarations). All samples were timely transported, under reverse cold chain at 4°C, to INRB at Kinshasa for storage at -20°C until processing.

### Cell lines and virus isolation

Stool samples received at the national reference laboratory for poliomyelitis at INRB were processed according to WHO recommended routine procedures as described in the polio laboratory manual [61]. Briefly, chloroform-treated and clarified 20% stool suspensions (weight/volume) were prepared from stool specimens and inoculated onto monolayered Human rhabdomyosarcoma (RD) and murine L20B (a derivative of murine L cells expressing the PV human receptor) cells. In contrast to AFP patients from who two stool specimens collected 48 h apart, only one stool specimen from each healthy child was systematically inoculated onto Human larynx epidermoid carcinoma (HEp-2c) cell in addition to RD and L20B cell cultures. Cell cultures in tubes were maintained in Dulbecco’s modified Eagle’s medium (D-MEM) supplemented with 2% fetal calf serum and 2 mM L-glutamine at 36°C. Infected tubes were observed daily for the appearance of cythopatic effect (CPE). Positive cultures were harvested and stored at -20°C while negative cultures were observed for five days and re-passaged onto a new monolayer culture for additional five days. Isolates harvested from RD and HEp-2c cell cultures were systematically inoculated on L20B cell cultures and the resulting suspected PV isolates were further typed as described below [61, 62]. Isolates showing CPE only on RD and/or HEp-2c, and not on L20B cell lines, were considered as NPEVs and were characterized as described below.

### Virus isolates from patients with Acute Flaccid Paralysis

#### Non-polio Enterovirus (NPEV) isolates

A total of 150 NPEV isolates derived from AFP patients originating from the Kasai Oriental (120 isolates) and Maniema (30 isolates) provinces from 2008 to 2012 were available for this study.

#### Poliovirus isolates

A collection of 91 Sabin-like isolates from AFP patients in 2008–2010 including 46 Sabin type 1 (38 from Kasai Oriental and 8 from Maniema), 25 Sabin type 2 (16 from Kasai Oriental and 9 from Maniema) and 20 Sabin type 3 (15 from Kasai Oriental and 5 from Maniema), was available for this study. These OPV related isolates were routinely identified by ITD [61–63].

On the other hand, 54 type 2 cVDPVs isolates obtained from 3, 7, 3 and 41 AFP patients respectively from the provinces of Equateur in 2010, Maniema in 2010, Kasai Occidental in 2008–2010 and Katanga in 2008–2012 were also considered for this study. All cVDPV isolates were identified both by ITD and sequence analysis of the full-length VP1 protein coding gene as previously reported [13, 61–64]. Although most cVDPV isolates analysed in this study originated from the Katanga province, field survey could not be carried out in Katanga because of acute security concerns during the study period.

Biosafety and biosecurity measures during transport, processing and storage of virus isolates were implemented according to the WHO-specified standards described in the ‘‘Polio laboratory manual” [61].

### RNA extraction and gene amplifications

Viral RNA were extracted and purified from 140 μL of infected cell culture supernatants using QIAamp Viral RNA Mini Kit according to the manufacturer’s instructions (Qiagen, Courtaboeuf, France).

### Molecular characterization of polioviruses

In order to evaluate the accuracy of the routine IDT assays for the detection of cVDPVs, full-length VP1 sequences of PV isolates classified as Sabin-like following ITD assays were determined using previously reported type-specific primers [28]. Each resulting VP1 sequence was compared with the homologous sequence of the homotypic reference OPV strains. Since a proportion of cVDPVs isolates included in this study, especially those of the 2012 outbreak, were not considered in the original report about VP1 characterization [13], full-length VP1 sequences of all cVDPV isolates were determined with the same methodology. Then the VP1 sequence variability was assessed by phylogenetic analysis as described below.

To search for potential OPV/non-polio EV-C recombinants, having non-polio EV-C related 3D^pol^ sequences without VP1 sequence divergence compared to corresponding reference OPV strains, we performed a Restriction Fragment Length Polymorphism (RFLP) assay. The RFLP assay used 4 distinct restriction enzymes (HinfI, DpnII, DdeI, and RsaI) to digest a 929-pb amplicon spanning the 3’-end of the 3D^pol^ and the entire 3’-UTR region of all Sabin-like isolates confirmed by VP1 sequence analysis. RT-PCR amplification and digestion analysis were carried out as previously described [65].

### Molecular characterization of non-polio enteroviruses

A 750-base pairs DNA fragment encompassing the 3’-end of the VP3 capsid region and the 5’-half of the VP1 capsid region of NPEV isolates was amplified using a reverse transcription-nested polymerase chain reaction (RT-nPCR) as previously described [27, 66]. To resolve the ambiguity specifically associated to the molecular typing of some EV-C types [41], complete sequence of the VP1 gene was determined for all EV-C isolates identified from their partial VP1 sequences. Overlapping amplicons, encompassing the complete VP1 gene, were generated by RT-PCR using EV-C specific primers reported elsewhere [27, 66].

Virus isolates that were refractory to the pan-enterovirus RT-nPCR targeting the VP3-VP1 genes were successfully amplified with a sensitive RT-nPCR technique amplifying a portion of 300–400 pb at the 5’-end of the VP1 gene the EV genome [67].

### Amplification of non-structural regions of de genome of Enterovirus C including cVDPVs

In order to investigate the extent of recombination among EV-C, a previously reported strategy [27] was used to amplify three additional regions (5’UTR, 2C^ATPase^ and 3D^pol^) of the viral genome of all cVDPV and non-polio EV-C isolates. Resulting amplicons were processed as described below.

### Sequencing and sequence analysis

Amplicons were analyzed using agarose gel electrophoresis. Depending on the presence of a single or multiple bands on the gel, amplicons were either directly purified with QIAquick PCR Purification kit or gel-isolated and purified using QIAquick Gel Extraction kit (Qiagen, Courtaboeuf, France) following the manufacturer’s protocol.

Resulting amplicons were subjected to direct sequencing using the BigDye terminator v3.1 kit (Applied Biosystems) and an ABI Prism 3140 automated sequencer (Applied Biosystems). The sequencing of each amplicon was performed in both directions using semi-nested PCR primers. Sequence data obtained using the ABI PRISM kits were viewed, assembled and edited using the CLC Main Workbench 5.7.2 software (CLC bio, Aarhus, Denmark). Consensus sequences were submitted to databases with the following accession numbers: MK310554 to MK310568 and MK310788 to MK310984 respectively for the VP1 and VP3-VP1 sequences of NPEVs; MK310569 to K310641, MK310642 to MK310714 and MK310715 to MK310787 respectively for the 2C^ATPase^, 3D^pol^ and 5’UTR sequences of cVDPVs; MK310985 to MK311038 for the full-length VP1 sequences of cVDPVs; MK311039 to MK311132 for the full-length VP1 sequences of Sabin-like PVs.

### Molecular typing of non-polio enteroviruses (NPEVs)

Consensus nucleotide sequence corresponding to the 5’-half or complete VP1 capsid gene of each NPEV isolate was analyzed by pairwise comparison with the homologous sequences of databases available prototype strains. As previously reported [28, 68, 69], scores were established for each strain according to nt and amino acid (aa) identity with homotypic and heterotypic strains. Virus types were supported by nt and aa identity with the closest sequence above the type assignment thresholds (75 and 85% nt and aa identity respectively). Field isolates were considered to belong to the same serotype as the closest prototype strain according to the results of pairwise comparisons of nt and aa sequences, as previously reported [68, 69].

### Phylogenetic analyses

Multiple sequence alignments were carried out for each data set using the CLC Main Workbench 5.7.2 software. A phylogenetic tree was inferred from each resulting nt sequence alignment by the Maximum-likelihood (ML) algorithm implemented in the MEGA version 7.7.1 software under the best nt substitution model estimated with the Smart Model Selection software based on the Bayesian Information Criterion; transition/transversion (Ts/Tv) ratio and ML base composition was estimated from empirical datasets [70–73]. Phylogenetic trees of the EV-A76 and EV-C were estimated using the most complex General Time-Reversible model of nucleotide substitution with proportion of invariable sites plus gamma-distributed rate heterogeneity with 4 rate categories (GTR+G+I). The phylogenetic tree for the EV-D111 data set was estimated using the T92+G model of nucleotide substitution which allow a discrete gamma distribution of among-site rate variation with 5 rate categories.

For the study of recombination among EV-C strains, 5’UTR, 2C^ATPase^ and 3D^pol^ sequences based phylograms were inferred from specific nt alignments using the ML algorithm implemented in the MEGA version 7.7.1 software under the best-fit nucleotide substitution model estimated with the Smart Model Selection program implemented in phyML [70–73]. Original analysis of 5’UTR, VP1, 2C^ATPase^ and 3D^pol^ sequences were more exhaustive including all studied nt sequences and all available homologous sequences from cVDPVs and EV-C originating from other epidemiologic contexts worldwide. Then most database available sequences featuring no peculiar relationships with the studied isolates were removed in order to improve the clarity and readability of the phylograms.

In all cases, alignment gaps were pairwisely removed from each sequence pair in the datasets and the reliability of the tree topologies was estimated by bootstrap analysis with 1,000 pseudo-replicate datasets.
